# New Property of Albumen

**Published:** 1874-11

**Authors:** 


A New Property of Albumen.—An interesting observation, and one which may in time develop some very important practical results, was recently announced to the French Academy of Sciences by MM. Mathieu and Unbane. * * * When the serum of blood is completely freed from gas, an albuminous liquid is obtained, which will not coagulate, even though it is heated to the boiling point of water. Further than this, they assert that carbonic acid is the agent to which the property of coagulability by heat is to be ascribed; and they find that 100 parts by volume of albumen contain nominally 75 to 85 volumes of carbonic acid; from 2 to 3 volumes of oxygen, and from 3 to 5 of nitrogen. By depriving the albumen, with the aid of the air-pump, of these gases, the property of coagulation is destroyed, but it can be restored by simply passing into it the necessary quantity of carbonic acid. The albumen so deprived of its gases behaves in all other respects towards reagents like ordinary albumen.

